# The social brain in the classroom: insights from the SELF study on neural correlates of adolescent motivation, belonging, and learning

**DOI:** 10.3389/fnhum.2026.1830571

**Published:** 2026-05-21

**Authors:** Diana Raufelder, Frances Hoferichter

**Affiliations:** School Pedagogy, Educational Science, University of Greifswald, Greifswald, Germany

**Keywords:** adolescence, educational neuroscience, neuroeducational model, school belonging, socio-emotional learning, teacher-student relationships

## Abstract

Adolescence is characterized by heightened sensitivity to social relationships, emotional experiences, and evaluative contexts, making schools a central developmental environment for motivation, learning, and brain maturation. Although educational research has long emphasized the importance of belonging, teacher-student relationships, and socio-emotional learning, and neuroscience has identified adolescence as a sensitive period for social-affective brain systems, these lines of research remain only loosely connected. As a result, socio-emotional school experiences are rarely examined in relation to their underlying neural mechanisms. This article synthesizes findings from the socio-emotional learning factors (SELF) study and introduces an integrative neuroeducational model of the social brain in the classroom. SELF represents an interdisciplinary research approach that combines educational science, psychology, and developmental and social neuroscience to investigate how adolescents’ socio-emotional experiences at school—such as belonging, social exclusion, teacher–student relationships, and socio-motivational orientations—are associated with psychological processes and neural systems supporting self-referential processing, socio-emotional salience, feedback learning, and cognitive control. Building on evidence from both behavioral and neuroimaging research, including longitudinal and cross-sectional studies, the proposed model conceptualizes learning in adolescence as a socially embedded process that is psychologically mediated and reflected in neural processes during a period of heightened developmental sensitivity and plasticity. Rather than assuming linear brain–behavior relationships, the model emphasizes reciprocal interactions between classroom social ecology, motivational and affective processes, and neural mechanisms. By integrating multiple levels of analysis, the SELF neuroeducational model advances a non-reductionist perspective on educational neuroscience. It provides a framework for theory-driven research and highlights socio-emotional classroom conditions—particularly belonging and relational quality—as foundational for adolescents’ motivation, learning, and socio-emotional development.

## 1 Introduction

Adolescence is a pivotal developmental period characterized by profound changes in social relationships, motivational orientations, and brain maturation (Blakemore, 2008, 2010; Bukowski et al., 2011; Harter, 1999). During these years, young people increasingly define their self-concept in relation to significant others—particularly peers and teachers—while simultaneously developing greater autonomy and self-regulatory capacities (Blakemore, 2008; Blakemore and Mills, 2014; Crone and Fuligni, 2020; Steinberg, 2008). Schools constitute one of the most influential social environments in adolescence, as they provide not only cognitive learning opportunities but also a dense network of social interactions, evaluations, and emotionally salient experiences. These experiences are central to students’ engagement, wellbeing, and academic development (Eccles and Roeser, 2011; Schlesier and Raufelder, 2024).

Educational and psychological research has long emphasized the importance of socio-emotional factors such as school belonging, supportive teacher-student relationships, and motivation for successful learning (Gillen-O’Neel and Fuligni, 2013; Raufelder, 2018; Roorda et al., 2011). Students who feel socially connected and emotionally secure at school tend to show higher intrinsic motivation, and better academic outcomes, whereas experiences of exclusion, social stress, or evaluative threat are associated with anxiety, disengagement, and maladaptive learning behaviors (Hoferichter et al., 2022; Korpershoek et al., 2020; Osterman, 2000; Roorda et al., 2011; Ryan and Deci, 2017). Recent integrative approaches further highlight that emotions and social relationships are not independent influences but are deeply intertwined, jointly shaping students’ everyday experiences of teaching and learning (Raufelder and Schlesier, 2026; Schlesier and Raufelder, 2024). Despite this robust body of evidence, socio-emotional processes in school are still often examined without explicit reference to the underlying neural mechanisms that support social, emotional, and motivational development during adolescence.

In parallel, advances in developmental and social neuroscience have demonstrated that adolescence represents a sensitive period for brain systems involved in self-referential processing, emotion regulation, and cognitive control, marked by heightened plasticity and increased responsiveness to socio-emotional experience (Crone and Dahl, 2012; Fuhrmann et al., 2015; Pfeifer and Peake, 2012). This perspective is complemented by theoretical accounts describing adolescence as a phase of social re-orientation, during which peers and other significant social partners, as well as evaluative social contexts, become increasingly salient for affect, motivation, and self-processing (Nelson et al., 2005). Brain regions such as the medial prefrontal cortex, precuneus, amygdala, insula, and anterior cingulate cortex undergo substantial functional and structural reorganization during this phase and are highly responsive to social experiences, including acceptance, rejection, feedback, and emotional cues from significant others (Blakemore, 2008, 2010; Raufelder et al., 2021a,b; Sebastian et al., 2011). Importantly, these neural systems form the basis of what is commonly referred to as the “social brain,” which integrates social information into self-related cognition, affective processing, and goal-directed behavior (Blakemore, 2012a). However, much of this neuroscientific research has been conducted in laboratory settings that remain only loosely connected to the everyday realities of schooling and classroom life.

This separation has contributed to a persistent gap between neuroscience and educational research. While educational scholars increasingly call for interdisciplinary approaches that consider brain development in real-world learning contexts, neuroeducational research has also been criticized for oversimplifying brain-behavior relations or for offering limited relevance for educational practice (Ansari and Coch, 2006; Bowers, 2016; Howard-Jones, 2014). At the same time, commentary and review work in educational neuroscience argues that neural and psychological levels of explanation can be complementary, particularly when research is theory-driven and context-sensitive (Howard-Jones et al., 2016; Thomas et al., 2019; Thomas and Arslan, 2024). Recent contributions in the field further emphasize the need for empirically integrative frameworks that connect neural mechanisms with socio-emotional and motivational processes in educational settings (Hoferichter and Raufelder, 2025; Thomas et al., 2019; Thomas and Arslan, 2024). What is still lacking, however, is a programmatic body of research that systematically links adolescents’ socio-emotional experiences in school with both functional and structural aspects of brain development and embeds these findings within established educational theories.

The socio-emotional learning factors (SELF) study adopts an interdisciplinary research perspective that bridges educational science, psychology, and neuroscience, with a specific focus on the school context and its relation to students’ brain development. The SELF study comprises a broader interdisciplinary line of research including multiple empirical studies that combine questionnaire-based, qualitative, structural MRI, and functional MRI approaches (e.g., Böhme et al., 2017; Golde et al., 2019; Raufelder et al., 2015, 2016, 2021a,b; Romund et al., 2017). Following a methodologically pluralistic approach, SELF combines two-wave questionnaire data, neuroimaging measures, and qualitative interviews to investigate how socio-emotional experiences emerging from relationships with teachers and peers shape adolescents’ motivation, learning processes, and neural development. In doing so, the study positions the classroom as a socially and emotionally embedded environment in which brain development and learning unfold in close interaction. In this sense, the SELF study complements and extends recent theoretical models that conceptualize socio-emotional school experiences as dynamic, relational, and context-sensitive processes (Raufelder and Schlesier, 2026; Schlesier and Raufelder, 2024).

Across a series of studies using longitudinal and cross-sectional design, the SELF study combines behavioral assessments, motivational profiles, and neuroimaging data to investigate neural systems supporting self-referential processing, emotion regulation, feedback-based learning, and cognitive control in adolescence. By focusing on school-related experiences such as belonging, social exclusion, teacher-student relationships, and socio-motivational orientations, SELF explicitly situates learning-related neural development within the social ecology of the classroom. This approach allows for a more nuanced understanding of how social experiences at school become integrated into neural processes that are directly relevant for learning, motivation, and socio-emotional development.

The aim of the present article is to synthesize findings from the SELF study and to outline a neuroeducational perspective on the social brain in the classroom. Specifically, we integrate evidence on adolescents’ neural processing of self and others, emotion processing in teacher–student relationships, socio-motivational orientations, and cognitive control, and embed these processes within the social ecology of the classroom.

To avoid conceptual ambiguity, it is important to distinguish between (a) the SELF study as an interdisciplinary, multi-method empirical approach, (b) the broader assumptions that guided its design, and (c) the integrative neuroeducational SELF model proposed in the present article. The SELF study was originally developed to investigate socio-emotional learning in school contexts across multiple levels of analysis. The model presented here does not precede the empirical studies in its current form. Rather, it represents a theoretically refined synthesis of empirical findings across the SELF studies.

Across this body of work, social classroom experiences—including relationships with teachers and peers as well as experiences of social belonging and exclusion—emerge as key contextual conditions that are closely intertwined with adolescents’ motivational, emotional, and learning-related processes. Building on this synthesis, we propose an integrative neuroeducational model of socio-emotional learning factors (SELF) that conceptualizes learning in adolescence as a socially embedded process unfolding through reciprocal interactions between classroom social experiences, psychological processes, and developing neural systems within a sensitive developmental phase. By grounding educationally relevant constructs in neural processes—without reducing learning to brain activity alone—this contribution seeks to advance a balanced and empirically informed dialogue between neuroscience and education.

## 2 The SELF framework: an interdisciplinary neuroeducational perspective

To further clarify the conceptual structure of the present contribution, we distinguish between the SELF study and the integrative neuroeducational SELF model proposed in this article. The SELF study refers to the broader interdisciplinary line of empirical studies combining questionnaire, qualitative, and neuroimaging approaches to investigate socio-emotional learning in school contexts. The integrative model presented here builds on this study of research and synthesizes its findings into a more explicit neuroeducational framework. Thus, the model should be understood as a conceptually refined synthesis rather than as a fully specified *a priori* framework that guided each individual study in its present form.

The SELF study was conceptualized to advance an interdisciplinary understanding of socio-emotional learning in adolescence by integrating educational science, psychology, and neuroscience with a clear focus on the school context. Rather than treating classroom learning as primarily cognitive process, the SELF study is grounded in the premise that adolescents’ learning, motivation, and self-development are embedded in their everyday relational and emotional experiences with teachers and peers. Accordingly, the central aim is to explain how socio-emotional experiences in school—such as belonging, exclusion, social evaluation, and relationship quality—are linked to motivational orientations and learning processes, and how these associations are reflected in functional and structural aspects of brain development.

### 2.1 Core assumptions

SELF builds on three interconnected assumptions: First, socio-emotional experiences in school are not peripheral but constitutive for learning and development in adolescence. Feelings of belonging, or exclusion shape students’ engagement, stress responses, and willingness to invest effort—often through subtle relational cues in teacher-student and peer interactions (Osterman, 2000; Raufelder et al., 2014). This premise aligns with integrative educational perspectives emphasizing that emotions and relationships jointly structure classroom experiences and adaptation over time (Schlesier and Raufelder, 2024).

Second, adolescents differ systematically in how strongly their motivation is tied to teachers and peers (Jagenow et al., 2015). SELF therefore adopts a person-centered perspective on motivation, assuming that socio-motivational orientations (i.e., the degree to which motivation depends on teacher or peer relationships) represent meaningful intra- and interindividual differences with implications for learning behavior and emotional functioning (Raufelder et al., 2013a,c).

Third, the developing brain provides a complementary level of analysis for understanding why socio-emotional classroom experiences matter, particularly during adolescence as a sensitive period for social-affective and self-related processing (Blakemore, 2012a; Raufelder, 2018). This sensitivity is closely linked to heightened neural plasticity, as adolescence has been conceptualized as a developmental window in which brain systems are especially responsive to environmental and social input (Fuhrmann et al., 2015). Research in developmental social neuroscience indicates that responses to social evaluation, acceptance, and rejection are supported by neural systems implicated in self-referential processing, salience detection, and regulatory control (Crone and Dahl, 2012; Pfeifer and Peake, 2012; Sebastian et al., 2011), including cortical midline structures such as the medial prefrontal cortex and precuneus, as well as regions implicated in salience detection and affective processing such as the amygdala and anterior insula. These systems are thus plausible biological pathways linking social classroom experiences with motivation and learning.

### 2.2 Key constructs: socio-emotional school experiences, motivation, and learning

Within SELF, socio-emotional school experiences are conceptualized as relationally embedded and context-sensitive processes that unfold in interactions and are interpreted by adolescents in light of their needs, goals, and self-concept (Hoferichter et al., 2018). To capture these processes, the framework focuses on (a) relationship quality with teachers and peers (e.g., support, emotional security, evaluative threat), (b) experiences of belonging versus exclusion, and (c) motivational orientations that reflect socio-motivational dependence versus independence.

Motivational orientations are treated as more than stable traits; they are understood as patterns of meaning-making that shape how adolescents respond to feedback, interpret teacher emotions, and regulate effort and engagement (Raufelder et al., 2013a,b). In this sense, the SELF perspective complements broader integrative models of socio-emotional school experiences by adding an explicit neuroeducational layer and by linking motivational profiles to learning-related neural processes (Schlesier and Raufelder, 2024).

### 2.3 Methodological approach: a multi-method design grounded in the school-context

A defining characteristic of SELF is its methodological pluralism, combining two-wave questionnaire data with neuroimaging and qualitative approaches to examine developmental phenomena at multiple levels of analysis. This multi-method strategy is crucial for a neuroeducational perspective that aims to remain anchored in the realities of schooling while also leveraging neuroscientific tools to illuminate mechanisms that are otherwise difficult to observe. In other words, the framework does not treat brain measures as replacements for educational constructs but as complementary evidence that can strengthen (or constrain) theoretical interpretations of socio-emotional learning in school contexts.

More specifically, the questionnaire component of the SELF study assessed key socio-emotional and motivational constructs relevant to adolescents’ school experiences. These included perceived school belonging and social exclusion, the quality of teacher–student and peer relationships, socio-motivational orientations (i.e., the extent to which motivation depends on teachers or peers), as well as indicators of academic self-concept, emotional wellbeing, and school-related stress. The use of two-wave data further allowed for the examination of developmental changes and associations over time between these constructs.

The qualitative component complemented these measures by capturing adolescents’ subjective interpretations of their social and emotional experiences in school. Semi-structured interviews focused on how students perceive and make sense of relationships with teachers and peers, experiences of inclusion and exclusion, and emotionally salient classroom situations. This approach provided a more nuanced understanding of how socio-emotional experiences are interpreted and integrated into students’ motivational and self-related processes.

These behavioral and self-report measures were systematically linked to neuroimaging paradigms targeting self- and other-referential processing, emotional reactivity to socially relevant cues (e.g., teacher expressions), feedback-based learning, and cognitive control. By combining these methods, the SELF study enables a trinagulative analysis of how socio-emotional classroom experiences are reflected in psychological processes and neural systems. Across studies, participants were adolescents in secondary school, with age ranges varying slightly depending on the specific sample and measurement wave, typically covering early to mid-adolescence.

A particular strength of the SELF study lies in the way neural measures are anchored in school-relevant experiences and constructs. Rather than relying solely on abstract or decontextualized laboratory tasks, several neuroimaging paradigms were explicitly linked to educationally meaningful stimuli and processes, such as self- and other-evaluations involving teachers and peers, emotional responses to teacher expressions, and feedback-based learning in evaluative contexts. In addition, neural indicators were systematically related to adolescents’ self-reported classroom experiences, including belonging, social exclusion, and socio-motivational orientations. This multi-method integration enhances the ecological interpretability of the findings by allowing neural processes to be understood in relation to adolescents’ everyday experiences in school contexts.

### 2.4 Conceptual positioning within contemporary theory development

Finally, the SELF framework was designed to be compatible with and extend emerging theory that conceptualizes socio-emotional experiences in education as dynamic, adaptive, and relationally co-constructed (Schlesier and Raufelder, 2024). In this regard, SELF can be positioned as a programmatic neuroeducational complement to the socio-emotional school experiences adaptation (SESEA) model, which provides a broader theoretical account of how emotions, social relationships, and the experience of learning and teaching are interwoven across educational actors and contexts (Raufelder and Schlesier, 2026). At the same time, SELF is compatible with the integrative framework of belonging (Allen et al., 2021), which conceptualizes belonging as a multidimensional and relational process shaped by competencies, opportunities, motivations, and perceptions, as well as with the model of reciprocal situated teacher-student relationships (Trauernicht et al., 2025), which emphasizes the dynamic, bidirectional, and context-embedded nature of classroom relationships. In addition, SELF provide empirical support for the model of student well-being (S-WELL-B) (Hoferichter, under review), which highlights the importance of fostering safe, connected, empowered, and balanced environments for students in order to enhance student wellbeing at multiple levels. While these frameworks provide broader conceptual accounts of socio-emotional experience, belonging, and teacher-student relationships in educational settings, SELF contributes converging empirical evidence—particularly through neuroimaging—on how specific socio-emotional school experiences and socio-motivational orientations are reflected in adolescents’ developing neural systems.

From a theoretical perspective, findings from the SELF study both strengthen and constrain existing interpretations of socio-emotional learning in school contexts. On the one hand, they provide converging support for theoretical approaches that conceptualize learning as socially embedded and relationally constituted, such as models of socio-emotional school experiences, belonging, and reciprocal teacher–student relationships (see Hagenauer and Raufelder, 2022; Raufelder and Schlesier, 2026; Trauernicht et al., 2025). The SELF findings extend these perspectives by demonstrating that such processes are not only psychologically meaningful but are also reflected in neural systems supporting self-referential processing, socio-emotional salience, and adaptive learning.

On the other hand, the findings constrain interpretations that treat socio-emotional factors as peripheral or secondary to cognitive aspects of learning. The consistent associations between classroom social experiences, motivational processes, and neural functioning suggest that socio-emotional processes are integral to how adolescents interpret, engage with, and regulate learning in school contexts. At the same time, the SELF study does not support reductionist accounts that prioritize neural explanations over psychological or contextual ones. Instead, the results highlight the need for integrative frameworks that consider social, psychological, and neural processes as dynamically interrelated.

## 3 Neural evidence from the SELF study: converging findings on motivation, belonging, and learning

The SELF study provides converging evidence that adolescents’ learning-related motivation and self-development are tightly intertwined with socio-emotional experiences in school and their neural correlates. Across functional and structural neuroimaging paradigms, SELF studies consistently highlight brain systems involved in (a) self- and other-referential processing, (b) socio-emotional salience and affective reactivity to teacher cues, and (c) feedback-based learning and cognitive control. Importantly, these neural processes are interpreted within the school context, where social evaluation, belonging, and teacher-student relationships are central and developmentally salient (Crone and Dahl, 2012). Across the studies reviewed in this section, the term “neural systems” refers to functionally interconnected brain regions that support specific aspects of socio-emotional and learning-related processing. In the context of the SELF study, this primarily includes (a) cortical midline structures involved in self-referential processing (e.g., medial prefrontal cortex and precuneus), (b) regions implicated in socio-emotional salience and affective processing (e.g., amygdala and anterior insula), and (c) prefrontal–striatal circuits supporting feedback-based learning, cognitive control, and adaptive behavior.

### 3.1 Self- and other-referential processing: the adolescent self-concept as socially embedded

A first line of evidence within the SELF study addresses adolescents’ neural processing of self-related information in relation to close and educationally relevant others. Using functional MRI and trait-evaluation paradigms, these studies investigated how adolescents’ self-referential processing and the quality of their relationships with friends and teachers map onto their neural structures. In these paradigms, participants evaluated whether trait adjectives applied to themselves, a close friend, or a teacher, allowing for direct comparisons of neural activation associated with self- and other-referential processing across socially relevant targets. Across these tasks, self-referential processing consistently engaged cortical midline structures, particularly the medial prefrontal cortex (mPFC) and the precuneus—regions widely implicated in self-related thought and social cognition.

Crucially, neural activation patterns varied systematically as a function of the reference person. In particular, evaluations of the self were associated with the strongest activation in ventromedial regions of the mPFC. Evaluations of friends elicited highly overlapping activation patterns, particularly within the mPFC and medial posterior parietal regions, whereas evaluations of teachers—although socially familiar and educationally significant—were associated with comparatively weaker activation in these self-related regions (Romund et al., 2017). This graded pattern suggests that the adolescent self-concept is neurally organized along a continuum of social closeness and personal relevance, with peers occupying a particularly central position during this developmental phase. Thereby, self-concept refers to individuals’ perceptions and beliefs about themselves, encompassing perceived attributes, roles, and characteristics that can be organized across different domains (Crone and Pfeifer, 2024).

Beyond these general patterns, SELF findings indicate that individual differences in socio-emotional experiences are reflected in adolescents’ neural self-other architecture. In a subsequent study, perceived loneliness—a marker of social disconnection—was associated with altered neural activation during self-, friend-, and teacher-related evaluations. Higher loneliness was linked to reduced activation in self-referential regions during self-evaluation, which in turn was associated with a lower academic self-concept (Golde et al., 2019). These results directly connect adolescents’ subjective social experiences in school with neural processes underlying the formation of academic self-views.

Recent longitudinal evidence from the SELF study further extends these findings by demonstrating that socially embedded self-referential processing is linked to developmental changes in academic self-concept across adolescence. Rodriguez Buritica et al. (2025) showed that both behavioral indicators of school performance and neural activation during evaluations of educationally relevant others predicted changes in adolescents’ academic self-concept over time. While cortical midline structures were primarily engaged during self-evaluations, activity in posterior midline regions, particularly the precuneus, during evaluations of teachers and peers was associated with trajectories of academic self-concept development. This highlights that neural processing of significant others is not merely concomitant to self-related cognition but contributes to how academic self-concepts evolve during adolescence.

Taken together, these findings indicate that adolescents’ self-related cognition is not neurally isolated but embedded within a social frame that includes peers and teachers as salient reference points. Moreover, variability in social experiences –such as loneliness –and in the neural processing of significant others is systematically linked to differences in academic self-concept and its development. These results align with broader developmental social neuroscience research identifying adolescence as a sensitive period for neural systems supporting self-evaluation, social comparison, and responses to social evaluation (Blakemore, 2012a; Sebastian et al., 2011).

*Take-home messages*

- Adolescents’ self-referential processing is neurally embedded in a social framework that includes peers and teachers, with peers showing particularly strong overlap with self-related neural activation (Romund et al., 2017).
- Individual differences in socio-emotional experience, such as loneliness, are associated with altered neural processing of the self and educationally relevant others, linking classroom social experiences to the neural basis of the academic self-concept (Golde et al., 2019).
- Longitudinal evidence indicates that neural processing of teachers and peers predicts developmental changes in adolescents’academic self-concept, underscoring the social embeddedness of self-concept development in school contexts (Rodriguez Buritica et al., 2025).

### 3.2 Belonging and exclusion: structural brain development in socio-emotional integration systems

A second line of evidence within the SELF study addresses whether school-related social experiences are associated with structural brain development during adolescence. In a longitudinal structural MRI study, we examined adolescents’ perceived school belonging and social exclusion in relation to gray matter volume (GMV) changes over a one-year period. These measures were derived from adolescents’ self-reports and linked to longitudinal structural MRI data using voxel-based morphometry, allowing the investigation of how individual differences in perceived social experiences are associated with changes in brain structure over time. Importantly, voxel-based morphometry revealed a specific association for social exclusion, but not for school belonging, with trajectories of cortical development in the left anterior insula (Raufelder et al., 2021a,b).

Adolescents who reported higher levels of social exclusion at school showed a smaller decrease in GMV in the left anterior insula from mid- to late adolescence. Given that normative brain development during this period is characterized by widespread gray matter reduction (Blakemore, 2012b), this pattern suggests a delay or alteration in typical maturational processes rather than accelerated development. No comparable associations were observed for school belonging, underscoring the specificity of exclusion-related experiences for structural brain development.

The anterior insula is a key region involved in socio-emotional salience detection, interoceptive awareness, and the integration of affective signals. From a neuroeducational perspective, these findings are highly relevant because they indicate that chronic experiences of social exclusion in the school context are associated with variability in the maturation of neural systems supporting socio-emotional integration. Importantly, these results should not be interpreted in a deterministic sense, nor do they imply direct causal effects of school experiences on brain structure. Rather, they suggest that adverse social experiences at school co-occur with altered neurodevelopmental trajectories in regions that are central for emotional processing and social functioning during adolescence.

*Take-home messages*

- Perceived social exclusion at school –but not school belonging –is associated with longitudinal changes in gray matter volume in the left anterior insula during adolescence (Raufelder et al., 2021a,b).
- Higher social exclusion is linked to a reduced normative decrease in gray matter volume, suggesting delayed or altered maturation in a region central to socio-emotional salience and integration.
- These findings support neuroeducational models that emphasize the developmental relevance of adverse social classroom experiences, while cautioning against simplistic or deterministic interpretations of brain-school relations.

### 3.3 Teacher-student relationships and affective processing: neural sensitivity to teacher cues

A third line of evidence within the SELF study addresses the affective meaning of teacher-student relationships and how emotional cues from teachers are processed in the adolescent brain. In an fMRI study using emotional facial expressions of teachers, adolescents’ neural responses were examined in relation to their socio-motivational relationships with teachers and their experience of academic anxiety. During the task, adolescents viewed teachers’ facial expressions depicting different emotional states, allowing the assessment of neural responses to socially evaluative and affectively salient cues in a school-relevant context. Results showed that stronger amygdala activation in response to teachers’ negative facial expressions (e.g., anger or disapproval) was associated with higher test anxiety and with less supportive socio-motivational teacher-student relationships (Raufelder et al., 2015).

This finding is neuroeducationally relevant because the amygdala plays a central role in processing affective salience and threat-related social cues. In classroom contexts, teachers’ emotional expressions function as highly salient social-evaluative signals, particularly during adolescence, when sensitivity to evaluation is heightened (Frenzel et al., 2021). The SELF findings thus provide a mechanistic account of how the relational and emotional climate of teaching shapes students’ emotional responses in academic contexts, demonstrating how everyday teacher cues may contribute to anxiety-related experiences at school.

Importantly, earlier interdisciplinary work within the broader SELF framework has extended this perspective beyond test anxiety. Using emotional face-processing paradigms with both teacher and peer stimuli, Pöhland and Raufelder (2014) showed that heightened neural sensitivity to negative emotional cues from educationally relevant others was associated with adolescents’ feelings of loneliness, depressive symptoms, and stress. These findings suggest that neural reactivity to teachers’ and peers’ emotional signals reflects a more general sensitivity to socio-emotional threat and emotional vulnerability, rather than anxiety alone.

Taken together, these results indicate that teacher-student relationships are not only motivationally relevant but are deeply embedded in adolescents’ affective neural processing. Supportive relationships may buffer the emotional meaning of evaluative signals, whereas strained relationships may heighten the salience of negative cues, with implications for adolescents’ socio-emotional development in school contexts.

*Take-home messages*

- Teachers’ emotional cues are processed as salient social-evaluative signals, engaging affective neural systems linked to academic anxiety and emotional vulnerability (Raufelder et al., 2015; Pöhland and Raufelder, 2014).
- Individual differences in teacher-student relationships shape the emotional meaning of classroom cues and may function as risk or protective factors for adolescents’ socio-emotional development.

### 3.4 Feedback-based learning and socio-motivational orientations: neural signatures of dependence vs. independence

A fourth line of evidence within the SELF study links adolescents’ socio-motivational orientations to neural processes underlying feedback-based learning. In an fMRI study employing a probabilistic reversal learning paradigm, reinforcement learning parameters and prediction error-related brain activity were examined in relation to adolescents’ socio-motivational (in-)dependence, defined by the extent to which peers and teachers function as sources of motivation (Raufelder et al., 2016). In this task, participants learned to select between stimuli based on probabilistic feedback and had to flexibly adjust their choices when stimulus-outcome contingencies changed. This design allows for the estimation of individual learning rates as well as neural prediction error signals associated with feedback processing.

At the behavioral level, individual learning rates—reflecting the extent to which recent feedback shapes subsequent choices—distinguished socio-motivationally dependent adolescents from their independent peers. Higher learning rates, indicating rapid adjustment to feedback, were associated with socio-motivational dependence, whereas lower learning rates characterized socio-motivational independence. Importantly, these differences were not related to overall task performance, but to distinct learning styles in response to feedback.

At the neural level, prediction error-related activation in prefrontal and striatal regions further differentiated motivational profiles. Greater prediction error-related activity in the right prefrontal cortex predicted a higher likelihood of belonging to a peer-and-teacher-dependent motivational type, consistent with the role of prefrontal regions in controlled, feedback-oriented learning processes. In contrast, stronger prediction error-related activation in the left associative striatum was associated with socio-motivational independence, suggesting a greater reliance on internally generated expectations and self-guided behavioral adaptation (Raufelder et al., 2016). These processes are particularly relevant in school contexts, where learning is continuously shaped by evaluative feedback from teachers and peers.

Within the SELF framework, these findings indicate that adolescents differ systematically in how their learning-related neural systems are tuned to external evaluation. Socio-motivational dependence is characterized by heightened sensitivity to feedback signals and greater engagement of control-related prefrontal systems, whereas socio-motivational independence is associated with neural patterns indicative of more autonomous learning regulation. In the school context, where learning is embedded in continuous social evaluation by teachers and peers, these differences provide a neuroeducational mechanism for understanding why some adolescents are particularly responsive to or vulnerable to evaluative classroom climates, while others rely more strongly on self-regulated learning processes.

*Take-home messages*

- Socio-motivational orientations are associated with distinct behavioral and neural signatures during feedback-based learning, particularly in learning rates and prediction error-related activation in prefrontal and striatal regions (Raufelder et al., 2016).
- These neural differences offer a neuroeducational explanation for variability in adolescents’ sensitivity to evaluative classroom environments and feedback practices.

### 3.5 Cognitive control and flexible adaptation: executive systems supporting adaptive learning

A final line of evidence relevant to the SELF framework concerns executive control processes that support flexible adaptation during learning. In a functional MRI study using a probabilistic reversal learning paradigm, adolescents’ ability to adapt behavior under changing reward contingencies was examined in relation to prefrontal activation patterns (Böhme et al., 2017). In this paradigm, participants had to adapt their choices in response to changing reward contingencies, requiring the inhibition of previously learned responses and the flexible updating of behavior based on feedback. The study demonstrated that successful strategy shifts and flexible adaptation were associated with stronger engagement of lateral and medial prefrontal regions implicated in cognitive control and goal-directed behavior.

Importantly, this paradigm was not explicitly designed to reflect a school context. Nevertheless, the findings are highly relevant for the SELF model because they highlight developmental variability in executive control systems that support adaptive learning more generally. Adolescents who showed greater reliance on goal-directed strategies exhibited stronger prefrontal activation and more efficient behavioral adaptation, whereas less flexible strategies were associated with reduced engagement of these control-related neural systems (Böhme et al., 2017).

Within the context of schooling, adaptive learning rarely occurs in emotionally neutral conditions. Classrooms are characterized by ongoing evaluation, performance pressure, and social comparison, all of which place demands on executive control and regulation. From a neuroeducational perspective, the SELF findings suggest that prefrontal control systems provide an important neural resource for maintaining flexible learning and strategy adjustment under such conditions. Although the reversal learning task does not directly model classroom interactions, it complements the SELF framework by illustrating how maturation of executive systems supports adolescents’ capacity to adapt learning behavior in environments that are cognitively and socially demanding.

*Take-home messages*

- Executive control systems, particularly prefrontal mechanisms, support flexible learning and strategy adjustment during adolescence (Böhme et al., 2017).
- Developmental differences in these systems are plausibly relevant for classroom adaptation, especially in learning situations characterized by evaluation, uncertainty, and changing demands.

### 3.6 Synthesis: converging pathways linking the social classroom to brain and learning

Across the different lines of evidence reviewed in Sections 3.1 to 3.5, findings from the SELF study converge on a coherent neuroeducational interpretation of learning in adolescence. Taken together, the results demonstrate that adolescents’ motivation, self-related development, and adaptive learning behavior are closely intertwined with how socially relevant information from the classroom is processed, evaluated, and regulated at the neural level.

First, studies on self- and other-referential processing show that the adolescent self-concept is neurally embedded in a social framework that includes peers and teachers as salient reference points, with individual differences in social connectedness shaping neural self-other representations and academic self-concept development (Section 3.1). Second, longitudinal evidence indicates that adverse social experiences at school—specifically social exclusion—are associated with variability in the maturation of neural systems involved in socio-emotional integration, particularly the anterior insula, highlighting the developmental relevance of negative social classroom experiences (Section 3.2). Third, research on teacher-student relationships demonstrates that teachers’ emotional cues engage affective salience systems, such as the amygdala, and are linked to students’ anxiety and emotional vulnerability, underscoring the affective significance of everyday classroom interactions (Section 3.3).

Complementing these social-affective pathways, findings on socio-motivational orientations and feedback-based learning reveal that adolescents differ in how strongly their learning-related neural systems are tuned to external evaluation, with distinct behavioral and neural signatures of sensitivity to feedback (Section 3.4). Finally, work on cognitive control and flexible adaptation highlights executive systems as an enabling resource for adaptive learning, particularly in contexts characterized by uncertainty and evaluative demands (Section 3.5).

Together, these findings support a neuroeducational framework in which learning during adolescence emerges from reciprocal interactions between the social classroom environment, psychological meaning-making, and developing neural systems. Rather than treating the “social brain” as an add-on to cognitive learning processes, findings from the SELF studies suggest that social and emotional signals are integral to how adolescents engage with schooling, regulate their behavior, and develop as learners.

## 4 Toward an integrative neuroeducational model of socio-emotional learning

Taken together, the findings from the SELF study point beyond isolated associations between social classroom experiences, psychological variables, and neural activation patterns. Instead, they converge on a coherent pattern in which adolescents’ learning-related motivation and engagement emerge from reciprocal interactions between classroom social experiences, psychological processes, and developing neural systems within a phase of heightened developmental sensitivity. Building on this synthesis, we propose an integrative neuroeducational model that conceptualizes learning in adolescence as a socially embedded and dynamically interconnected process.

### 4.1 From empirical findings to an integrative framework

Across the SELF studies, socio-emotional experiences in school—such as social evaluation, teacher-student relationships, and experiences of belonging and exclusion—are consistently associated with adolescents’ motivational orientations, emotional responses, and learning-related behavior. At the same time, these experiences are linked to functional and structural characteristics of neural systems involved in self-referential processing, socio-emotional salience detection, feedback-based learning, and cognitive control. Importantly, the pattern of findings across the SELF study does not support a linear or reductionist interpretation in which classroom experiences are simply “translated” into neural responses. Rather, the evidence points to reciprocal and dynamic interactions between social experiences, psychological processes, and neural functioning as they unfold in everyday school contexts. Importantly, the integrative SELF model is not intended as a direct representation of a single empirical study or as a fully specified framework that preceded the research program in its current form. Rather, it reflects a conceptual integration of findings across the different SELF sub-studies, making explicit the reciprocal relations between social classroom experiences, psychological processes, and neural systems.

Building on this synthesis, the proposed SELF neuroeducational model integrates three analytically distinct but interdependent levels of explanation: (a) the social ecology of the classroom, including relationships with teachers and peers as well as evaluative and emotional classroom climates; (b) psychological processes related to motivation, self-concept, affect, and regulation; and (c) neural systems supporting socio-emotional and learning-related processing. These levels are not conceived as hierarchically ordered or causally unidirectional. Instead, the model assumes that educational development emerges from their ongoing interaction over time. The learner is positioned at the center of this system, emphasizing that learning and development arise from continuous transactions between individuals and their social environments.

This integrative perspective is consistent with developmental-contextual and socio-cultural approaches in educational psychology, which conceptualize learning as embedded in dynamic interactions between persons and contexts (Eccles and Roeser, 2011; Lerner, 1991; Lerner et al., 2015). From a neurodevelopmental perspective, the model aligns with frameworks that highlight adolescence as a phase of heightened sensitivity to social and affective information alongside increasing demands on cognitive control and flexibility (Blakemore, 2008, 2010; Crone and Dahl, 2012; Fuhrmann et al., 2015). By bringing these perspectives together, the integrative neuroeducational model of socio-emotional factors (SELF) provides a conceptual framework for understanding how socio-emotional classroom experiences, psychological meaning-making, and neural development jointly shape learning during adolescence.

### 4.2 Structure and logic of the integrative neuroeducational model of socio-emotional learning (SELF)

**Figure 1:**
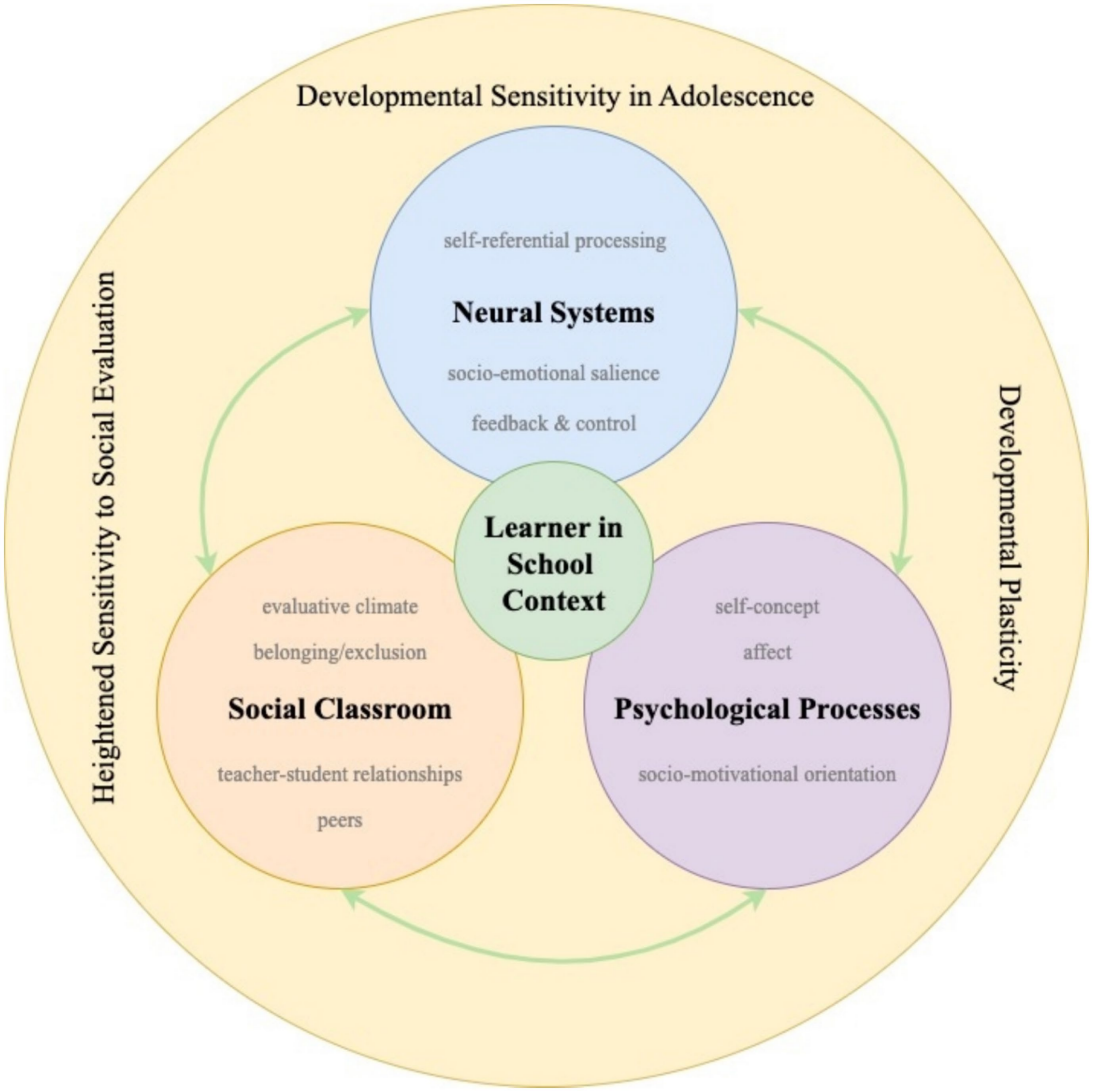
An integrative neuroeducational model of socio-emotional learning factors (SELF) in adolescence.

Figure 1 illustrates the structure and logic of the SELF integrated neuroeducational model. The organization of the model directly reflects the methodological design of the SELF study, which combines self-report measures of classroom experiences, person-centered analyses of socio-motivational orientations, and neuroimaging indicators to examine shared developmental phenomena from different but complementary levels of analysis. The model is therefore not intended to represent a causal sequence of effects, but to integrate distinct perspectives that have been empirically examined within the same research program.

At the contextual level, the model foregrounds the social classroom environment, including relationships with teachers and peers, experiences of social belonging and exclusion, and the evaluative and emotional climate of instruction. These social conditions constitute the experiential context within which adolescents encounter academic demands, interpret feedback, and engage in learning-related activities.

At the psychological level, the model captures processes through which classroom experiences acquire personal meaning for learners. These include socio-motivational orientations, school-related self-concept, and affective responses such as emotional security or anxiety in evaluative situations. Rather than functioning as mediators in a statistical sense, these processes are understood as dynamically intertwined with both classroom experiences and learning-related behavior, shaping how adolescents perceive, interpret, and respond to educational demands.

At the neural level, the model refers to systems supporting self- and other-referential processing, socio-emotional salience and integration, feedback-based learning, and executive regulation. Neural indicators are not conceptualized as causal drivers of learning, but as biological instantiations of processes that are simultaneously psychological and social in nature. In this sense, neural measures provide complementary evidence for mechanisms through which socio-emotional classroom experiences and psychological processes are embedded in the developing brain.

Crucially, the bidirectional arrows in the model indicate reciprocal influences rather than linear pathways. Classroom social experiences, psychological processes, and neural systems continuously shape and constrain one another over time. The learner is positioned at the center of the model to emphasize that learning and development emerge from these ongoing interactions within the social ecology of the classroom.

### 4.3 Core mechanisms linking social classroom experiences and learning

Within the integrative SELF framework, three interrelated mechanisms emerge that link social classroom experiences with adolescents’ motivation, learning-related behavior, and self-development. These mechanisms do not represent discrete pathways, but analytically distinguishable processes that are empirically supported across the SELF studies.

First, self-related learning processes in adolescence are neurally embedded in a social frame that includes educationally relevant others. Functional neuroimaging evidence demonstrates that adolescents’ self-referential processing systematically incorporates peers and teachers as reference points, with graded activation patterns reflecting social closeness and personal relevance (Romund et al., 2017). Individual differences in socio-emotional experience further shape this neural self–other architecture. In particular, loneliness has been linked to altered neural processing of self, friends, and teachers, with implications for adolescents’ academic self-concept (Golde et al., 2019). Longitudinal findings extend this mechanism by showing that neural processing of teachers and peers predicts developmental changes in academic self-concept across adolescence (Rodriguez Buritica et al., 2025). Together, these results indicate that social classroom experiences provide a reference structure through which academic experiences are integrated into self-related meaning-making.

Second, socio-emotional cues in the classroom calibrate adolescents’ sensitivity to evaluation and affective meaning. SELF findings show that teachers’ emotional expressions engage affective salience systems, particularly the amygdala, and are associated with students’ test anxiety and emotional vulnerability (Raufelder et al., 2015). Complementary evidence indicates that heightened neural sensitivity to negative emotional cues from teachers and peers is also linked to broader indicators of emotional distress, including loneliness, depressive symptoms, and stress (Pöhland and Raufelder, 2014). From this perspective, the emotional climate of instruction is not merely an accompanying feature of learning, but shapes how evaluative situations are perceived—either as supportive guidance or as socially threatening.

Third, adolescents differ in their socio-motivational orientations, which modulate how strongly learning processes are tuned to external evaluation versus internal standards and self-regulatory processes. Person-centered analyses within the SELF study link socio-motivational (in-)dependence to distinct behavioral and neural signatures during feedback-based learning, including differences in learning rates and prediction error–related activation in prefrontal and striatal regions (Raufelder et al., 2016). These findings suggest that motivational orientations reflect systematic differences in evaluation sensitivity, helping to explain why similar classroom environments may elicit divergent motivational and emotional responses across students.

Taken together, these three mechanisms illustrate how social classroom experiences, psychological meaning-making, and neural processes are tightly interwoven in adolescent learning. Rather than operating independently, they jointly shape how adolescents engage with academic demands, respond to evaluation, and develop as learners within socially and emotionally charged school contexts.

### 4.4 Developmental sensitivity and plasticity in adolescence

A central feature of the SELF neuroeducational model is its explicit grounding in adolescence as a developmental phase characterized by heightened sensitivity to social and emotional information. Developmental and social neuroscience research has consistently shown that adolescence involves substantial functional and structural reorganization of brain systems supporting self-referential processing, socio-emotional salience, and cognitive control (Blakemore, 2008; Crone and Dahl, 2012; Nelson et al., 2005; Sebastian et al., 2011). During this period, adolescents show increased responsiveness to social evaluation, acceptance, and rejection, rendering socio-emotional experiences particularly salient for motivational, emotional, and self-related development.

From a neuroeducational perspective, this heightened sensitivity is especially relevant in school contexts, where social evaluation is frequent and often unavoidable. Classroom interactions with teachers and peers expose adolescents to a dense stream of socio-emotional signals, including feedback, emotional expressions, and relational cues. The integrative SELF model builds on social neuroscience accounts of adolescent susceptibility to social evaluation by situating these processes within the concrete social ecology of the classroom, thereby linking developmental sensitivity to everyday educational experiences rather than laboratory-based social paradigms alone.

Empirical evidence from the SELF study supports this developmental interpretation with respect to adverse social experiences. Longitudinal structural MRI findings indicate that perceived social exclusion at school—but not school belonging—is associated with variability in trajectories of cortical development in the left anterior insula, a region implicated in socio-emotional salience and integration (Raufelder et al., 2021a,b). Adolescents reporting higher levels of exclusion showed a reduced normative decrease in gray matter volume over time, suggesting altered or delayed maturation rather than accelerated development. These findings do not imply deterministic effects of schooling on the brain; instead, they indicate that adolescence is a phase in which variability in negative social classroom experiences co-occurs with variability in neurodevelopmental pathways relevant for emotional regulation and learning-related functioning.

Importantly, the emphasis on developmental sensitivity in the integrative SELF model is complemented by the concept of developmental plasticity. Adolescence is not only a period of increased vulnerability to adverse social experiences, but also a phase in which supportive relational environments may buffer stress and support adaptive regulation (Hoferichter et al., 2021). While the SELF findings provide direct neurodevelopmental evidence primarily for the role of social exclusion, converging evidence from educational and developmental research suggests that emotionally secure classroom climates and supportive teacher-student relationships may function as protective contextual conditions during this sensitive period.

Taken together, this developmental perspective underscores that the relevance of socio-emotional classroom experiences is not static across the lifespan. Rather, their impact is amplified during adolescence due to heightened neural sensitivity and ongoing plasticity. By integrating this developmental dimension, the SELF integrated neuroeducational model highlights why schools—and particularly the quality of social relationships within them—constitute a crucial developmental context shaping adolescents’ motivation, socio-emotional functioning and engagement with learning.

### 4.5 Scope, limitations, and future directions

The proposed SELF integrative neuroeducational model is intended as a conceptual framework rather than a comprehensive or causal theory of learning. Its primary aim is to synthesize empirical findings from the SELF study and to provide a conceptual structure for understanding how social classroom experiences, psychological processes, and neural systems jointly shape adolescents’ learning-related motivation and adaptation.

Several limitations should therefore be acknowledged. First, the model does not seek to explain academic achievement or learning outcomes in a narrow performance-based sense. Instead, it focuses on motivational, emotional, and self-related processes that are foundational for learning engagement and developmental adaptation during adolescence. Second, although neural indicators are incorporated, the model does not assume direct or deterministic brain-behavior relationships. Neural systems are conceptualized as biological instantiations of socio-emotional and psychological processes, not as primary causes of educational outcomes. Accordingly, the integrative SELF model does not support brain-based prescriptions for teaching practice. Third, the model is explicitly grounded in adolescence and in the school context. Its assumptions and mechanisms cannot be readily generalized to early childhood, adulthood, or non-school learning environments without substantial theoretical refinement and empirical testing. Moreover, much of the available evidence within the SELF study is correlational in nature. While longitudinal designs provide insight into developmental trajectories, causal inferences regarding directionality and malleability remain limited. In addition, the SELF studies focus on a specific set of socio-emotional processes, particularly belonging, social exclusion, teacher–student relationships, and socio-motivational orientations. Other relevant factors identified in prior research—such as family context, broader peer dynamics, or classroom climate—were not systematically examined and should be considered in future work. Furthermore, although the samples capture important phases of secondary school development, they do not provide a comprehensive coverage of all adolescent substages, and findings may not generalize equally across early, middle, and late adolescence. Finally, the predominantly correlational nature of the data limits causal interpretation, and potential confounding factors—such as individual differences in personality, prior achievement, or mental health—cannot be fully ruled out.

At the same time, these limitations point to important directions for future research. Longitudinal and intervention-based studies are needed to examine whether changes in classroom social climates are accompanied by changes in psychological processes and neural indicators over time. The model further suggests that socio-motivational orientations may shape how adolescents respond to educational interventions, highlighting the importance of considering individual differences in sensitivity to social evaluation. Finally, advancing neuroeducational research requires continued efforts to integrate neuroimaging with ecologically valid assessments of classroom experiences, ensuring that neuroscientific insights remain theoretically grounded and educationally relevant.

Despite these limitations, the SELF neuroeducational model offers a coherent and empirically informed framework for conceptualizing how social classroom experiences become psychologically and biologically embedded during adolescence. As such, it provides a foundation for future research aimed at understanding—and ultimately supporting—adaptive learning and development in socially embedded educational contexts.

## 5 Educational implications: supporting learning and development in a sensitive phase

From a developmental and educational perspective, the SELF neuroeducational model underscores that adolescence is not only a phase of increasing academic demands, but also a sensitive developmental period in which social experiences and emotional classroom climates exert a particularly strong influence on both learning and broader developmental trajectories. Educational implications therefore cannot be reduced to instructional techniques or short-term performance outcomes. Rather, they concern how schools function as developmental contexts that either support or constrain adolescents’ social, emotional, and motivational development.

In this regard, the integrative SELF model aligns closely with stage-environment fit theory, which posits that developmental outcomes depend on the degree to which educational environments match adolescents’ changing socio-emotional needs (Eccles and Midgley, 1989; Eccles and Roeser, 2011). During adolescence, sensitivity to social evaluation, belonging, and relationships with peers and teachers increases markedly, while neural systems involved in social cognition, emotion regulation, and cognitive flexibility are still maturing (Blakemore, 2008, 2010; Bukowski et al., 2011; Harter, 1999). Classrooms thus become powerful developmental arenas in which relational experiences are not only psychologically meaningful but also biologically embedded.

From this perspective, social relationships and the emotions embedded within them are not secondary to academic learning but constitute a core developmental resource. Evidence from the SELF study and related research indicates that experiences of social exclusion and relational insecurity in school are associated with long-term risks for social functioning and wellbeing, and may co-occur with alterations in the maturation of neural systems supporting socio-emotional integration and cooperation (Blakemore, 2010; Chen et al., 2018; Raufelder et al., 2021a,b). Teachers and educators should therefore be aware that the social climates they co-construct in classrooms contribute not only to students’ learning engagement, but also to their socio-emotional and developmental trajectories.

Importantly, the SELF findings further highlight that adolescents differ substantially in how they respond to social-evaluative classroom contexts. Socio-motivational orientations shape how feedback, guidance, and autonomy are perceived and translated into learning-related behavior. Consistent with developmental contextualism (Lerner, 2006), these findings underscore that no single instructional approach fits all learners. Some adolescents benefit from clear guidance and frequent feedback, whereas others thrive in more autonomous and flexible learning environments. Supporting adolescent development therefore requires sensitivity to individual motivational styles and the creation of learning environments that allow for variability in engagement, regulation, and adaptation (Radel et al., 2010; Roth et al., 2007; Schweder and Raufelder, 2024a, 2024b; Soenens and Vansteenkiste, 2005).

Taken together, the educational implications of the SELF integrative model point toward a broadened understanding of teaching in adolescence. Being “brain-aware” does not mean applying neuroscientific techniques or prescriptions in the classroom, but recognizing that adolescents learn within a sensitive developmental phase in which social relationships and emotions play a central role (Jensen, 2005). Schools that foster relational security, autonomy-supportive climates, and opportunities for positive social engagement contribute not only to academic learning, but also to healthy social and emotional development—outcomes that are themselves foundational for sustained engagement and long-term educational success.

In conclusion, the SELF neuroeducational model provides a more comprehensive account of adolescent learning in school contexts than approaches that focus exclusively on social, psychological, or neural levels of analysis. By integrating these perspectives, the model highlights that socio-emotional classroom experiences are not peripheral influences but constitute core processes through which adolescents interpret, engage with, and regulate learning. In doing so, the SELF framework extends existing theories of socio-emotional learning by embedding them within a multi-level, developmentally sensitive account that captures the dynamic interplay between classroom context, psychological meaning-making, and neural development.

## Data Availability

The original contributions presented in the study are included in the article/supplementary material, further inquiries can be directed to the corresponding author.
